# Resting Cytosolic and Nuclear Reactive Oxygen Species (ROS) Are Regulated by the Basal Activity of ET-1 Receptors in Human Vascular Smooth Muscle Cells

**DOI:** 10.3390/ijms27062524

**Published:** 2026-03-10

**Authors:** Ghassan Bkaily, Rana Semaan, Danielle Jacques

**Affiliations:** Department of Immunology and Cell Biology, Faculty of Medicine and Health Sciences, Université de Sherbrooke, Sherbrooke, QC J1H 5N4, Canada

**Keywords:** ET-1, ET_A_ and ET_B_ receptors, homodimerized receptor, heterodimerized receptor, ROS, calcium, nucleus, cytosol, human vascular smooth muscle

## Abstract

Endothelin-1 (ET-1) is a potent vasoconstrictor that exerts its numerous biological actions through two receptors, ET_A_ and ET_B_. However, the implication and role of each receptor in ROS generation remain ambiguous. Previously, our group reported that blocking the basal activity of ET_A_ and ET_B_ receptors with their respective peptidic antagonists increased basal intracellular calcium (Ca^2+)^ levels, an effect inhibited by chelating extracellular Ca^2+^. Since a crosstalk between Ca^2+^ and reactive oxygen species (ROS) exists, the purpose of the present work was to investigate whether this increase in basal resting Ca^2+^ level induced by the blockade of ET_A_ and ET_B_ receptors is associated with an increase in resting ROS level. Our results showed that the basal activity of ET_A_ and ET_B_ receptors contributes negatively to the resting level of cytosolic and nuclear ROS, and that each receptor appears to act as the other’s physiological antagonist. Furthermore, our results showed that ET-1 receptor blockade increases ROS via a receptor insensitive to ET_A_ and ET_B_ receptor antagonists. This type of receptor could be the one reported by our group, ET_C_, or simply a heterodimeric ET_A_/ET_B_ receptor. Moreover, blocking the heterodimerized ET_A_/ET_B_ binding site is sufficient to unblock the physiological antagonism that each receptor exerts on the other. Furthermore, our results showed that blocking both ETA and ETB receptors, thereby preventing heterodimerization, prevented the increase in resting ROS, supporting the existence of a heterodimerized ET-1 receptor. Since human vascular smooth muscle cells (VSMCs) express only ETB receptors at the nuclear membrane, it is possible to suggest that nuclear ETB receptors are homodimers that regulate the resting nuclear ROS level. In conclusion, our results showed that the regulation of resting ROS levels by ET-1 and its receptors can be mediated by homodimerized and/or heterodimerized receptor activation; hence, the importance of developing drugs targeting this receptor type.

## 1. Introduction

### 1.1. Endothelin-1 (ET-1)

Originally isolated from the culture media of aortic endothelial cells by Yanagisawa and its group in 1988, ET-1 has a long-lasting and 10 times more potent vasoconstrictor activity than angiotensin II, which makes it a major regulator of blood pressure [1,2,3,4,5].

ET-1 is constitutively secreted at a basal level; nonetheless, its secretion can be stimulated by many mechanisms. ET-1 assists in cardiovascular homeostasis by controlling vascular tone and remodeling [5,6,7]. 

Endothelin exerts its physiological effects through two receptors: ET_A_ and ET_B_. ET_A_ and ET_B_ receptors are both found in endothelial cells [8,9,10,11] and VSMCs [3,9,12,13,14].

When activated, ET_A_ receptors lead to a slow-onset, sustained vasoconstriction. Activation of ET_B_ receptors on endothelial cells leads to vasodilatation [15,16] via the release of dilator mediators such as NO and PGI2, which act on smooth muscle cells; whereas activation of ET_B_ receptors on smooth muscle cells induces rapid vasoconstriction [16,17,18,19].

ET-1 can induce ROS signaling by activating nicotinamide adenine dinucleotide phosphate oxidase (Nox) via a Ca^2+^ dependent pathway [20,21,22] in VSMCs [23,24]. Endothelin-induced vasoconstriction seems to be reliant on the generation of reactive oxygen species [21,23], and Nox inhibition in rat arteries weakens ET-1-mediated vasoconstriction [25,26].

Interestingly, ROS is also known to increase ET-1 production. Hydrogen peroxide in human aortic VSMCs increased ET-1 levels [27], which were inhibited by antioxidants. However, the receptor through which ET-1 mediates its effect is still not known [28,29]. It seems that the blockage of both receptors with a dual ET_A_/ET_B_ antagonist can reverse the effect of ET-1 on ROS [30,31,32,33], which suggests that the effect of ET-1 on ROS could be mediated via a heterodimerization receptor [34,35,36]. This aspect remains a matter of debate.

### 1.2. ET-1 and Calcium

Tight regulation of Ca^2+^ homeostasis lies at the center of cardiovascular health and diseases, as it is the “master” of the excitation–contraction coupling process [3,37]. In VSMCs, the sustained increase in intracellular Ca^2+^ induced by ET-1 is mediated by both ET_A_ and ET_B_ receptors and occurs at both the cytosolic and nuclear levels [38]. This effect is not due to the activation of T-type or L-type Ca^2+^ channels, but due to the activation of the nifedipine-insensitive but isradipine-sensitive steady-state voltage-dependent resting-type R-type Ca^2+^ channel [38]. A close relationship between Ca^2+^ overload and ROS generation may account for ET-1’s effect on VSMCs, which needs to be verified.

### 1.3. Crosstalk Between Calcium and ROS

Calcium is one of the most important messengers in the cell; it regulates contraction, secretion, metabolism, gene expression, cell survival, and death [39]. Also, ROS are widely involved in physiological processes. In fact, Ca^2+^ and ROS signaling systems are intimately integrated, with several ROS-generating and antioxidant systems being Ca^2+^ dependent [40,41] and regulation of Ca^2+^ signals being redox-dependent [42]. Intracellular Ca^2+^ seems to regulate both ROS generation and ROS clearance, thereby shifting the redox state toward either a more oxidized or a more reduced state. Hence, the Ca^2+^ ions can increase ROS generation by enhancing the metabolism, and during this process, more electrons leak from the respiratory chain [42]. An increase in intracellular Ca^2+^ enhances ROS production [43]. Although the mechanism of Ca^2+^ induced mitochondrial ROS generation is not fully understood.

### 1.4. Endothelin Receptor Dimerization?

Similarly, ET-1 receptors have been suggested to form homo- or heterodimers [34]. ET-1 might act as a bivalent ligand, where its N-terminal binds to ET_A_ and its C-terminal end binds to ET_B_ [44,45]. The dissociation of the dimerized complex was observed after internalization, and ET-1 binding to ET_B_ stabilized the receptor at the plasma membrane [46,47]. To date, it is accepted that ET-1 increases ROS levels in VSMCs by activating NADPH oxidase [20,23,41]. However, the implication and role of each receptor in ROS generation remain ambiguous, in particular in human VSMCs. In addition, it is not known whether ET_A_ and/or ET_B_ receptors have a resting basal activity that regulates resting basal ROS generation, as well as upon their activation by ET-1.

Furthermore, previous results from our laboratory showed that the blockade of ET_A_ and ET_B_ receptors did not reverse the effect of ET-1 induced intracellular Ca^2+^ in human VSMCs [48]. Moreover, ROS resting levels differ between vascular endothelial and smooth muscle cells and are regulated by vasoactive hormones, such as ET-1 [49]. Thus, crosstalk may exist between ET-1, its receptors, intracellular Ca^2^, and ROS, and this may occur at the resting level. Thus, the aim of our study is to investigate the intracellular basal resting Ca^2+^ level blockade of basal activity of ET_A_ and ET_B_ receptors, as well as their activation by ET-1, which may regulate intracellular resting basal ROS homeostasis via heterodimerized ET_A_-ET_B_ receptor-induced Ca^2+^ influx through the R-type Ca^2+^ channel in human VSMCs, with a sensitivity to L-type Ca^2+^ channel blockers, such as nifedipine and verapamil.

## 2. Results

### 2.1. Contribution of the ET-1 Receptors ET_A_ and ET_B_ to the Basal ROS Level in hVSMCs

In this series of experiments, we wanted to verify whether endothelin receptors contribute to the determination of the resting level of ROS in hVSMCs.

#### 2.1.1. Effect of Blockade of the ET_A_ Receptors on the Resting ROS Level of hVSMCs

**Figure 1:**
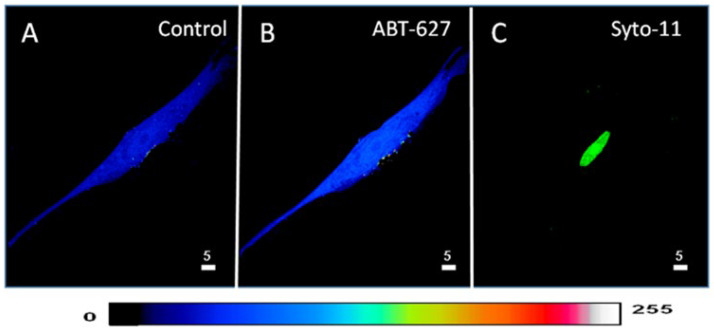
Effect of the blockage of the ET_A_ receptors on the basal ROS level in hVSMCs. (**A**) Real 3D image of hVSMCs loaded with ROS probe carboxy-H2DCFDA (DCF). (**B**) Blocking ET_A_ receptors using its nonpeptidic antagonist ABT-627 (10^−5^ M) led to an increase in the basal ROS concentration. (**C**) Staining of the nucleus by the live nucleic acid stain Syto-11. The pseudocolor scale illustrates fluorescence intensity corresponding to an arbitrary scale from 0 nm to 255 nm, where 0 represents the absence of fluorescence (black), and 255 represents the maximum fluorescence (white).

**Figure 2:**
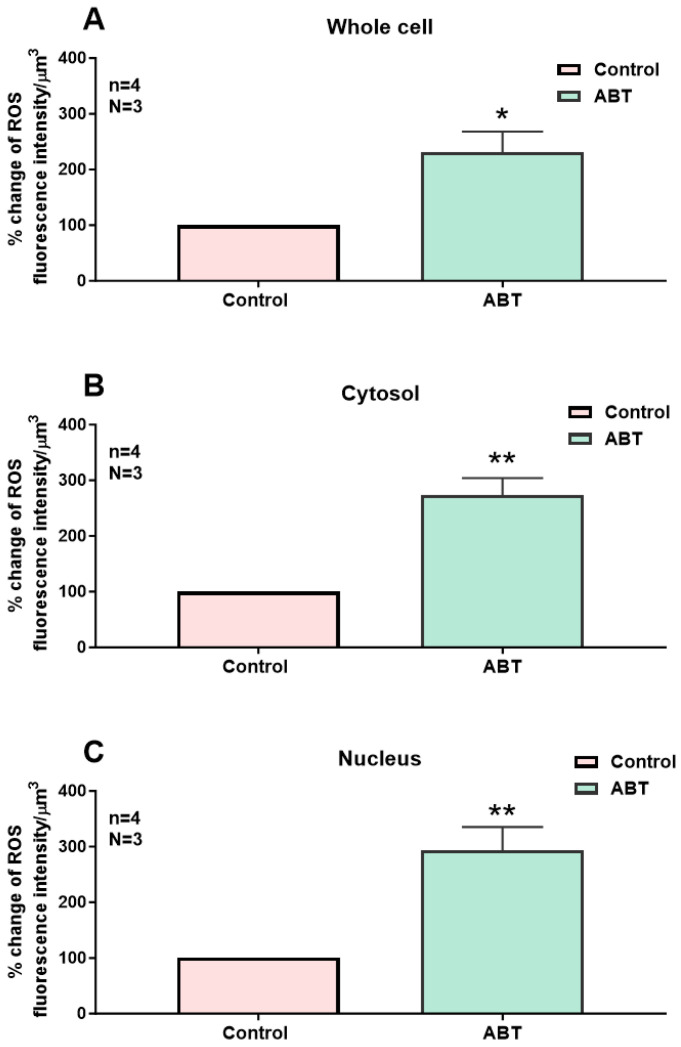
Effect of the nonpeptidic ET_A_ receptor antagonist ABT-627 on basal ROS concentration in hVSMCs. Effect of ABT-627 on basal ROS concentration in the whole cell (**A**), cytosol (**B**), and nucleus (**C**) of the hVSMCs. Values are expressed as mean ± SEM. N represents the number of different experiments, and n represents the number of cells. * *p* < 0.05, ** *p* < 0.01. The measurements were taken after reaching a steady state level.

In the first series of experiments, we determined whether blocking the basal activity of ETA receptors modulates the distribution and levels of total, cytosolic, and nuclear ROS in hVSMCs. Figure 1 shows an example, and Figure 2 summarizes the results. As shown in Figure 1, resting ROS is distributed homogeneously throughout the cell (Figure 1A). Superfusion with 10^−5^ M of the nonpeptidic specific ET_A_ antagonist ABT-627 induced an apparent sustained heterogeneous increase in resting intracellular ROS (Figure 1B). As shown in Figure 2, the increase in resting ROS following superfusion with an ET_A_ receptor antagonist was statistically significant at the whole-cell (Figure 2A, *p* < 0.05), cytosolic (Figure 2B, *p* < 0.001), and nuclear (Figure 2C, *p* < 0.001) levels.

#### 2.1.2. Effect of Blockade of the ET_B_ Receptors on the Resting ROS Level of hVSMCs

**Figure 3:**
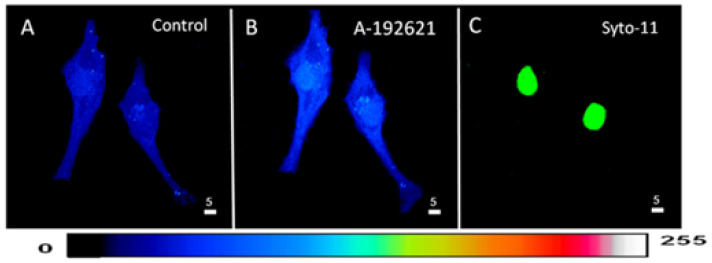
Effect of the blockage of the ET_B_ receptors on the basal ROS level in hVSMCs. 3D reconstitution images of cells loaded with ROS probe carboxy-H2DCFDA (DCF) (Scale bars 5 µm). (**A**) Basal ROS level. (**B**) Blocking ET_B_ receptors using its nonpeptidic antagonist ABT-627 (10^−5^ M) induced an increase in the basal ROS level. (**C**) Staining of the nucleus by the live nucleic acid stain Syto-11. The pseudocolor scale illustrates fluorescence intensity corresponding to an arbitrary scale from 0 nm to 255 nm, where 0 represents the absence of fluorescence (black), and 255 represents the maximum fluorescence (white).

Following superfusion with an ET_B_ receptor antagonist, the effect was statistically significant throughout the whole-cell (Figure 3A, *p* < 0.001), cytosolic (Figure 3B, *p* < 0.001), and nuclear (Figure 3C, *p* < 0.001) levels.

**Figure 4:**
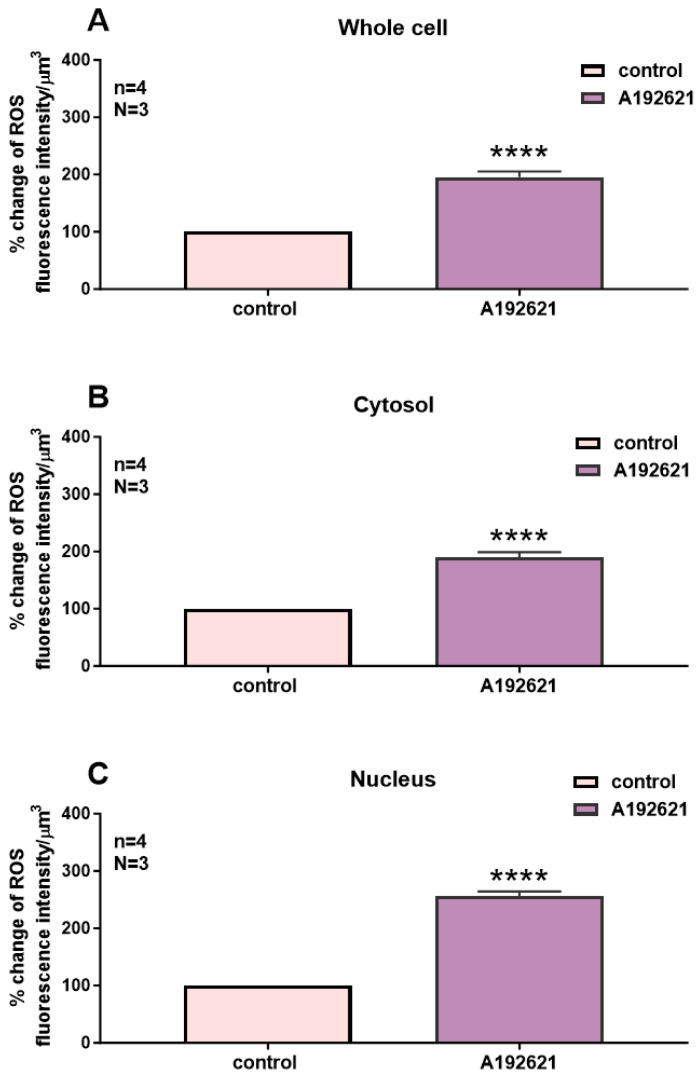
Effect of the nonpeptidic ET_B_ receptor antagonist A-192621 on basal ROS concentration in hVSMCs. Effect of ABT-627 on basal ROS concentration in the whole cell (**A**), cytosol (**B**), and nucleus (**C**) of the hVSMCs. Values are expressed as mean ± SEM. N represents the number of different experiments, and n represents the number of cells. **** *p* < 0.0001. The measurements were taken after reaching a steady state level.

In a second series of experiments, we verified whether the blockade of the basal activity of ET_B_ receptors modulates the distribution and levels of whole-cell, cytosolic, and nuclear resting ROS in hVSMCs. Figure 4 shows an example, and Figure 4 summarizes the results. As shown in Figure 4, apparent resting ROS is distributed homogeneously throughout the cell (Figure 3A). Superfusion with 10^−5^ M of the nonpeptidic specific ET_B_ antagonist A-192621 induced a sustained homogeneous apparent increase in resting intracellular ROS (Figure 3B).

#### 2.1.3. Effect of the Subsequent Blockage of Both Receptors ET_A_ and ET_B_ on the Resting ROS Levels in hVSMCs

**Figure 5:**
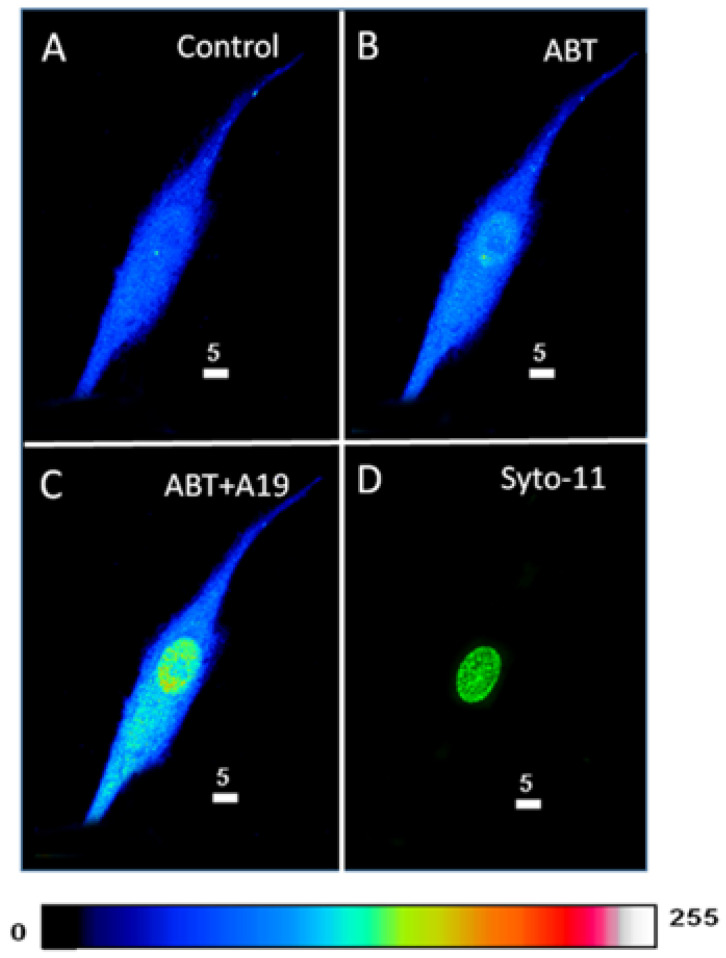
Effect of the subsequent blockage of both receptors ET_A_ and ET_B,_ on the basal ROS level in hVSMCs, shown via 3D reconstitution images of cells loaded with ROS probe (Scale bars 5 µm). (**A**) Basal ROS level. Addition of 10^−7^ M ABT-627 (**B**) led to an increase in basal ROS level in the cytosol and in the nucleus; however, addition of 10^−7^ M A-192621 to block the ET_B_ receptors (**C**) induced a further increase in ROS level in the nucleus but not in the cytosol. (**D**) The nucleus is stained using the live nucleic acid stain Syto-11. The pseudocolor scale illustrates fluorescence intensity corresponding to an arbitrary scale from 0 nm to 255 nm, where 0 represents the absence of fluorescence (black), and 255 represents the maximum fluorescence (white).

**Figure 6:**
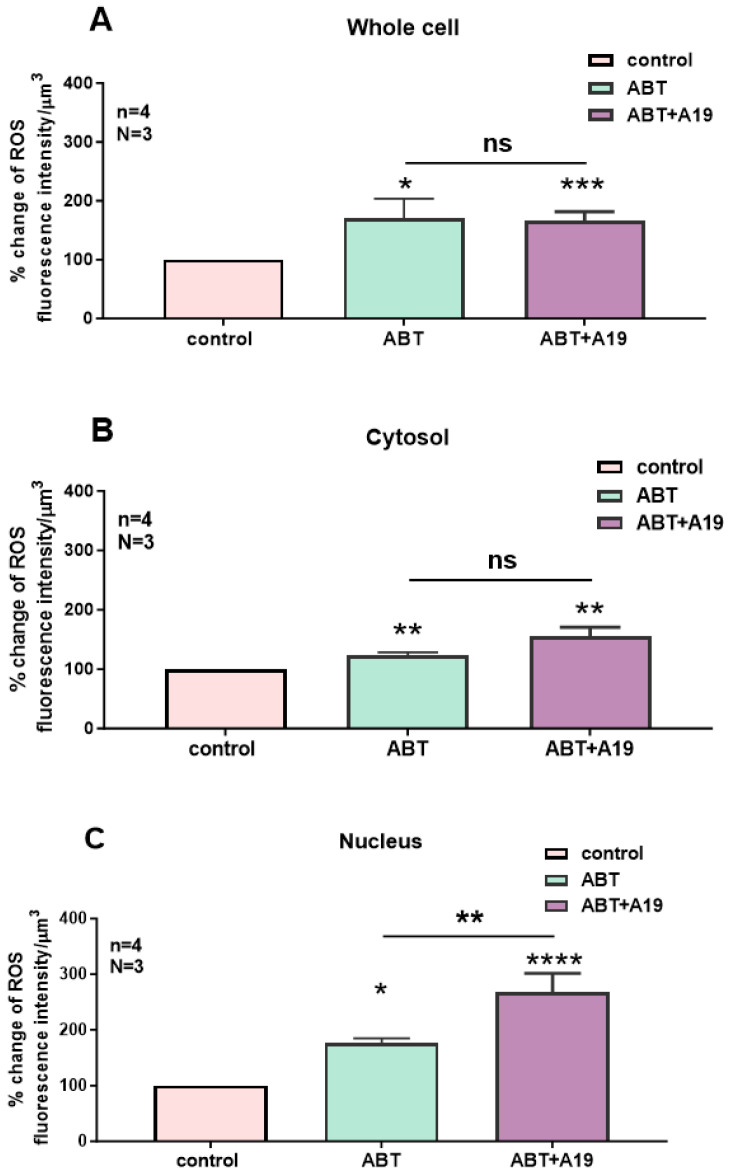
Effect of the nonpeptidic ET_A_ receptor antagonist ABT-627 and the subsequent addition of the nonpeptidic ET_B_ receptor antagonist A-192621 on basal ROS concentration in hVSMCs. Histograms showing measurements taken using confocal microscopy of the effect of the blockade of ET_A_ receptors, following the blockade of the ET_B_, on intracellular basal ROS concentration in the (**A**) whole cell, (**B**) cytosol, and (**C**) nucleus in VSMCs. Values are expressed as mean ± SEM. N represents the number of different experiments, and n represents the number of cells. * *p* < 0.05, ** *p* < 0.01, *** *p* < 0.001, **** *p* < 0.0001, ns: not significant.

In the last series of experiments, we determined whether blocking the basal activity of both ET_A_ and ET_B_ receptors modulates the distribution and levels of whole-cell, cytosolic, and nuclear resting ROS in hVSMCs. Figure 5 shows an example, and Figure 6 summarizes the results. As seen in Figure 5, apparent resting ROS is distributed homogeneously throughout the cell (Figure 5A). Superfusion with 10^−5^ M of the nonpeptidic specific ET_A_ antagonist ABT-627 induced a sustained heterogeneous apparent increase in resting intracellular ROS, which seems to be higher at the nuclear level when compared to that of the cytosolic level (Figure 5B). In the presence of the ET_A_ receptor antagonist, superfusion with 10^−5^ M of the nonpeptidic ET_B_ receptor antagonist induced a further apparent increase in cytosolic and nucleoplasmic resting ROS levels (Figure 5C). However, compilation of the results (Figure 6) shows that the increase in nucleoplasmic resting ROS induced by the ETB receptor antagonist is highly significant (Figure 6C, *p* < 0.01), without further increases in whole-cell and cytosolic resting levels of ROS (Figure 6A,B).

### 2.2. Effect of ET_A_ and ET_B_ Receptor Antagonist on ET-1 Induced Increase in Cytosolic and Nuclear ROS Levels in hVSMCs

ET-1 increases ROS levels in many cell types; however, the receptor type implicated is not known. In addition, whether the increase in resting ROS levels by ET-1 occurs at both the cytosolic and nuclear levels needs to be explored. In this series of experiments, we determined whether the effect of ET-1 was mediated via ET_A_ and/or ET_B_ receptors.

#### 2.2.1. Effect of ET-1 and the Subsequent Blockage of Its ET_A_ Receptor on ROS Level in hVSMCs

**Figure 7:**
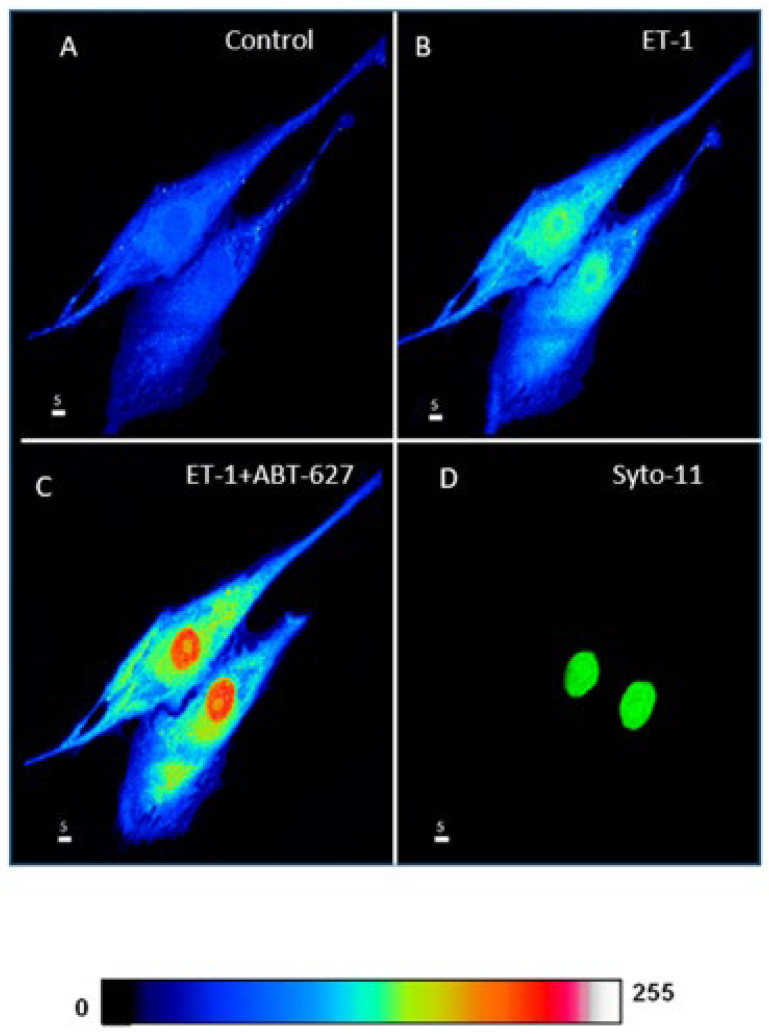
Effects of Endothelin 1 (ET-1) and nonpeptidic ET_A_ receptor antagonist ABT-627 on basal ROS concentration in hVSMCs (Scale bars 5 µm). (**A**) Real 3D image of hVSMCs loaded with ROS probe carboxy-H2DCFDA (DCF). Addition of ET-1 (10^−7^ M) (**B**) induced a sustained increase in ROS. (**C**) Blocking ET_A_ receptors using its antagonist ABT-627 (10^−5^ M) induced a further increase in ROS. (**D**) Staining of the nucleus by the live nucleic acid stain Syto-11. The pseudocolor scale illustrates fluorescence intensity corresponding to an arbitrary scale from 0 nm to 255 nm, where 0 represents the absence of fluorescence (black), and 255 represents the maximum fluorescence (white).

**Figure 8:**
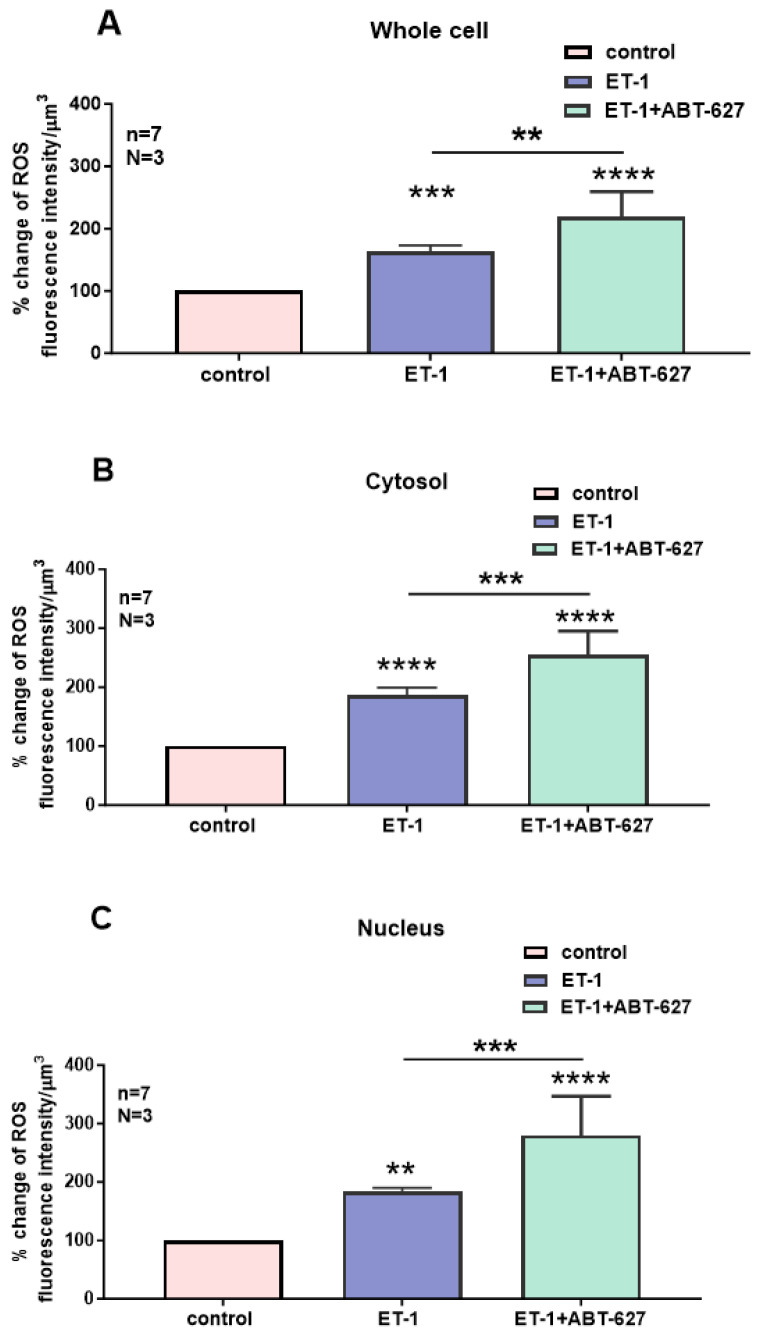
Effect of Endothelin 1 (ET-1) and ET_A_ receptor antagonist ABT-627 on basal ROS concentration in hVSMCs. Effects of ET-1 and ABT-627 on ROS concentration in the whole cell (**A**), cytosol (**B**) and nucleus (**C**) of VSMCs of a 35 years old woman. Values are expressed as mean ± SEM. N represents the number of different experiments, and n represents the number of cells. ** *p* < 0.01, *** *p* < 0.001, **** *p* < 0.0001. The measurements were taken after reaching a steady state level.

In the first series of experiments, we determined whether the increase in intracellular ROS induced by ET-1 could be reversed by blocking ET_A_ receptors. Figure 7 shows an example, and Figure 8 summarizes the results.

As shown in Figure 7, resting ROS is distributed homogeneously throughout the cell (Figure 7A). Superfusion with 10^−7^ M of ET-1 induced an apparent increase at both the cytosolic and nuclear levels, and this increase was heterogeneous throughout the cell. In the presence of ET-1, superfusion with the nonpeptidic specific ET_A_ antagonist ABT-627 induced a further apparent sustained heterogeneous increase in intracellular ROS that was visible at the nuclear level (Figure 7C). As seen in Figure 8, the increase in ROS levels following superfusion with ET-1 was significantly high in the whole-cell (Figure 8A, *p* < 0.001), cytosolic (Figure 8B, *p* < 0.001), and nuclear levels (Figure 8C, *p* < 0.01). Figure 8 also shows that in the presence of ET-1, blockade of ET_A_ receptors induced a further significant increase throughout the cell, including the whole-cell (Figure 8A, *p* < 0.01), cytosolic (Figure 8B, *p* < 0.001), and nuclear (Figure 8C, *p* < 0.001) levels.

#### 2.2.2. Effect of ET-1 and the Subsequent Blockage of Its ET_B_ Receptor on ROS Level in hVSMCs

**Figure 9:**
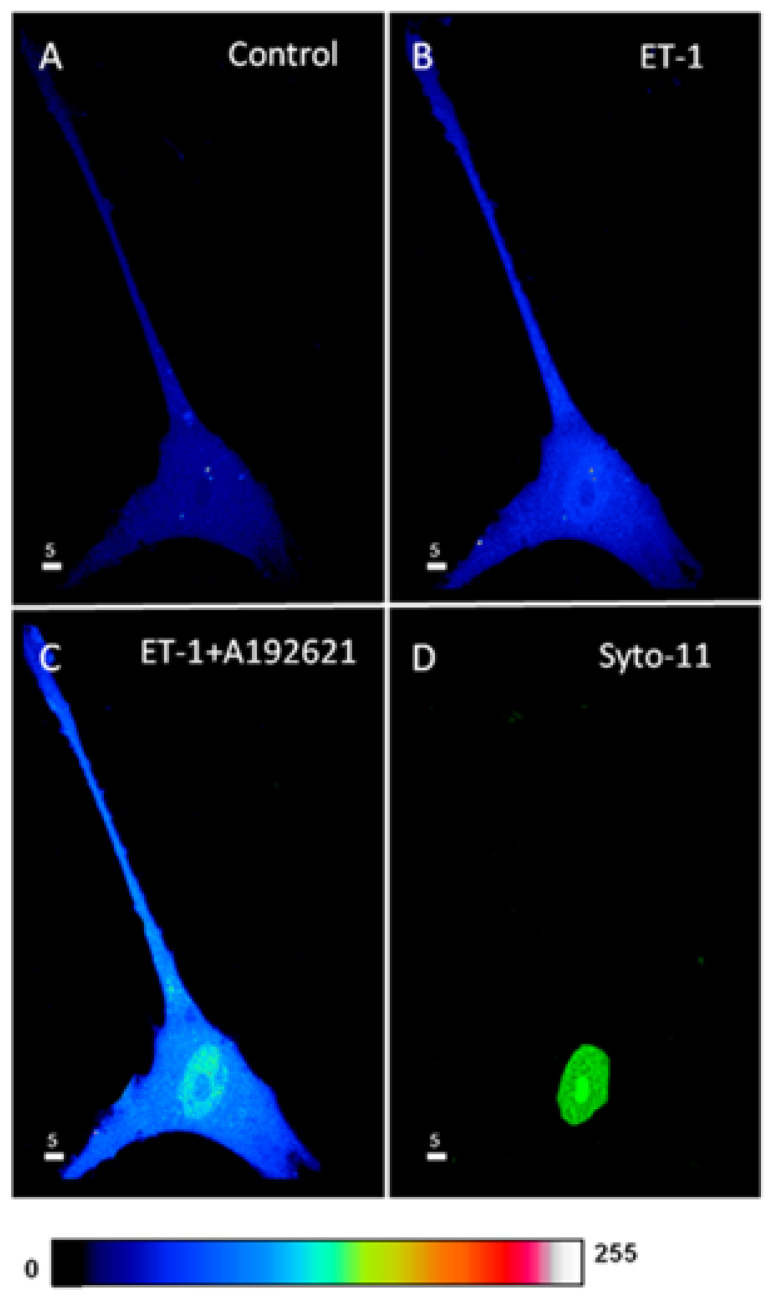
Effect of extracellular ET-1 and ET_B_ receptor antagonist A-192621 on basal ROS concentration in hVSMCs. Real 3D images of cells loaded with ROS probe (Scale bars 5 µm). (**A**) Basal ROS level. (**B**) Stimulation with ET-1 (10^−7^ M) increases ROS level. (**C**) Addition of ET_B_ antagonist A-192621 (10^−5^ M) did not reverse the effect of ET-1. (**D**) Labeling of the nucleus with Syto-11. The pseudocolor scale shows fluorescence intensity corresponding to an arbitrary scale going from 0 (black) nm to 255 nm (white).

**Figure 10:**
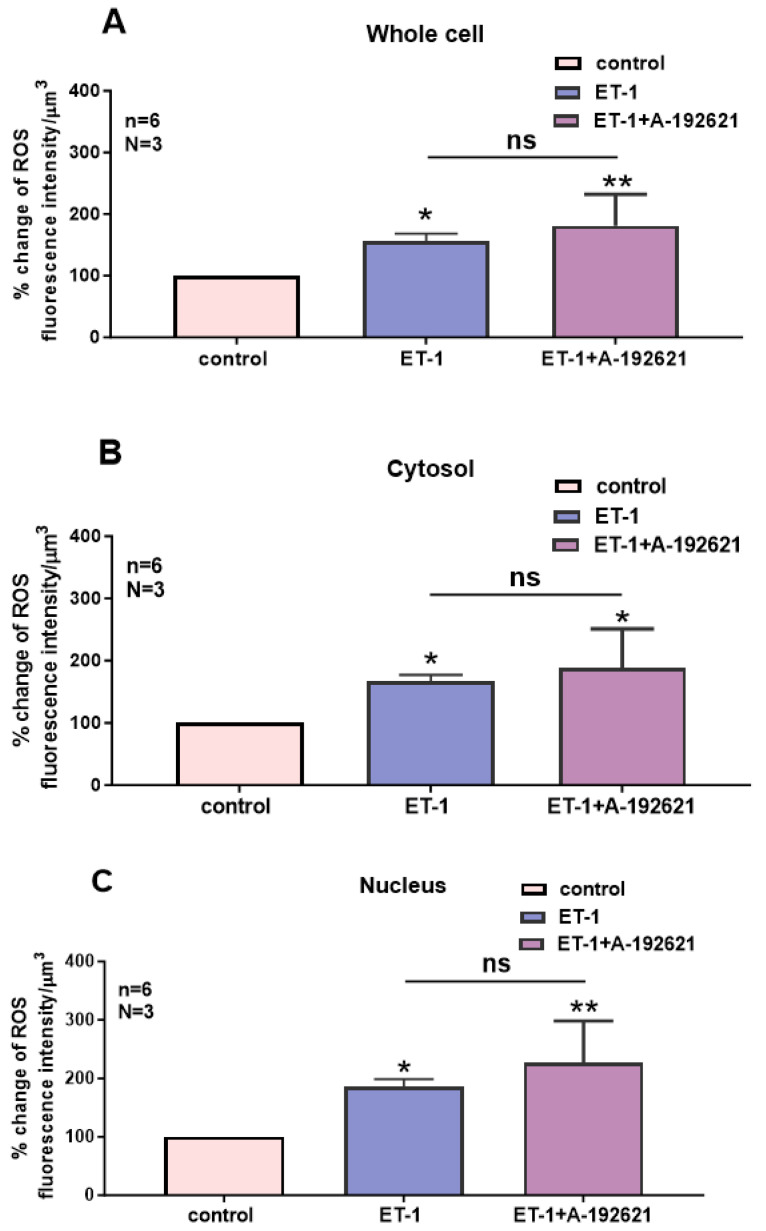
Effect of Endothelin 1 (ET-1) and ET_B_ receptor antagonist A-192621 on basal ROS concentration in hVSMCs. Effects of ET-1 and A-192621 on ROS concentration in the whole cell (**A**), cytosol (**B**), and nucleus (**C**) of the hVSMCs. Values are expressed as mean ± SEM. N represents the number of different experiments, and n represents the number of cells. * *p* < 0.05, ** *p* < 0.01, ns: not significant. The measurements were taken after reaching a steady state level.

In this series of experiments, we determined whether the increase in intracellular ROS by ET-1 could be reversed by blocking the ET_B_ receptors. Figure 9 shows an example, and Figure 10 summarizes the results. As shown in Figure 9, the resting ROS level, as expected, is distributed homogeneously throughout the cell (Figure 9A). Superfusion with 10^−7^ M of ET-1 induced an apparent increase at both the cytosolic and nuclear level and this increase was heterogeneous throughout the cell. In the presence of ET-1, superfusion with the nonpeptidic specific ET_B_ antagonist A-192621 seems to induce an apparent further sustained heterogeneous increase in intracellular ROS (Figure 9C). However, compilation of the results shown in Figure 10 demonstrates that the apparent further increase observed after ET_B_ receptor blockade was not statistically significant throughout the cell (Figure 10). This result strongly suggests that the effect of ET-1 on ROS levels is not mediated via ET_A_ or ET_B_ receptor activation, and that only ET_A_ receptor blockade further increases the ET-1-induced increase in ROS.

### 2.3. Effect of Preblockade of ET_A_ and ET_B_ Receptors and Glutathione on ET-1 Induced Increase in Cytosolic and Nuclear ROS in hVSMCs

**Figure 11:**
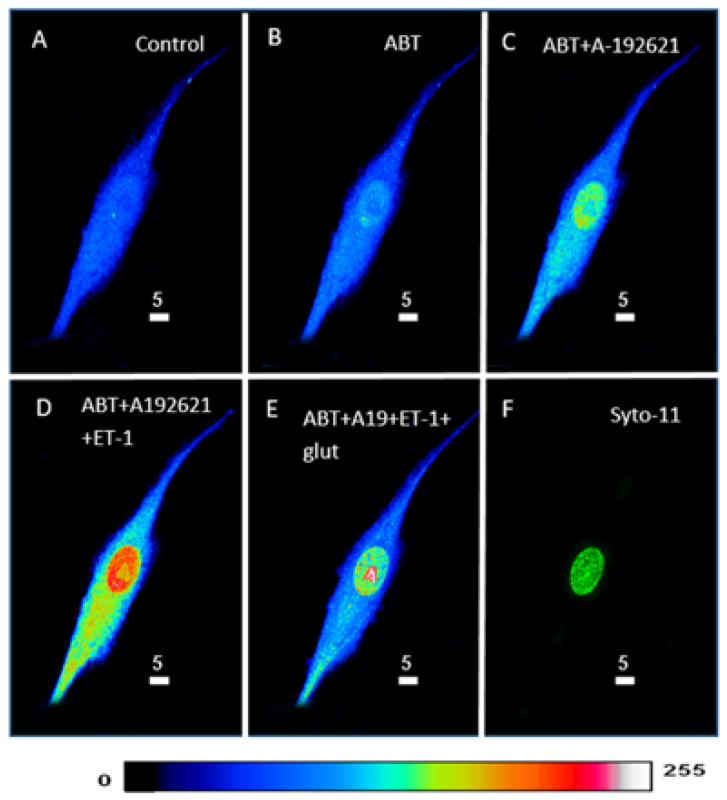
Effect of extracellular ET-1 on ROS concentration in the presence of ET_A_ antagonist ABT-627 and ET_B_ antagonist A-192621 in VSMCs. 3D reconstitution images of cells loaded with ROS probe (Scale bars 5 µm). (**A**) Basal ROS level. Addition of 10^−7^ M ABT-627 (**B**) and 10^−7^ M A-192621 (**C**) did not prevent ET-1 from inducing an increase in ROS level (**D**). This increase was reversed by the addition of (10 mM) glutathione (**E**). (**F**) The nucleus is stained using the live nucleic acid stain Syto-11. The pseudocolor scale illustrates fluorescence intensity corresponding to an arbitrary scale from 0 nm to 255 nm, where 0 represents the absence of fluorescence (black), and 255 represents the maximum fluorescence (white).

**Figure 12:**
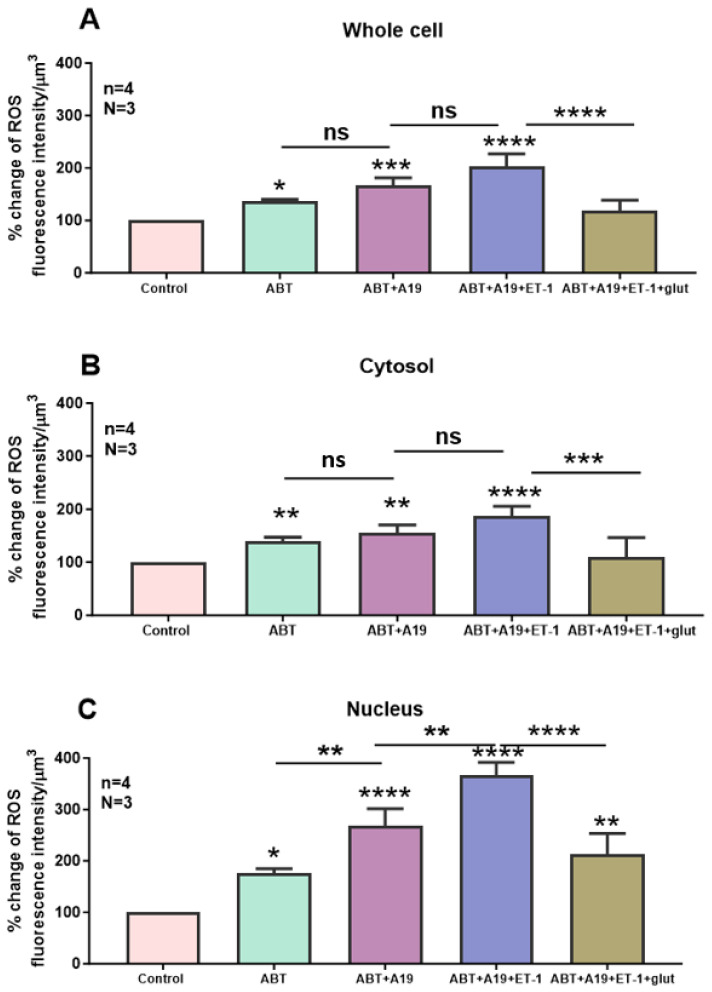
Effect of extracellular ET-1 on ROS concentration in the presence of ET_A_ antagonist ABT-627 and ET_B_ antagonist A-192621 in VSMCs. Histograms showing measurements taken using confocal microscopy of the effect of extracellular ET-1 on intracellular ROS concentration in the presence of ET_A_ antagonist ABT-627 and ET_B_ antagonist A-192621 in (**A**) whole cell, (**B**) cytosol, and (**C**) nucleus in VSMCs. Values are expressed as mean ± SEM. N represents the number of different experiments, and n represents the number of cells. * *p* < 0.05, ** *p* < 0.01, *** *p* < 0.001, **** *p* < 0.0001, ns: not significant.

In this series of experiments, we wanted to verify whether the ET-1-induced increase in intracellular ROS could be affected by the preblockade of ET_A_ and ET_B_ receptors and whether this effect could be reversed by increasing the antioxidant level of glutathione. Figure 11 shows an example, and Figure 12 summarizes the results. As can be seen in Figure 11B, the blockade of ET_A_ receptors induced an apparent increase in cytosolic and nuclear ROS levels (Figure 11B). As expected, in the presence of the ET_A_ receptor antagonist, superfusion with the ET_B_ receptor antagonist A-192621 did not further induce an apparent increase in basal ROS level (Figure 11C). As expected, in the presence of ET_A_ and ET_B_ receptor antagonists, superfusion with 10-7M ET-1 further increased the apparent level of ROS, particularly in the nucleus (Figure 11D). In the presence of the ET-1 and the ET-1 receptor antagonists, superfusion with 10 mM glutathione reversed the ROS level to near the level observed in the presence of the two antagonists of the ET-1 receptors (Figure 11E). As can be seen in Figure 12, and as expected, the blockade of the ET_A_ receptor induced a significant increase throughout the cell (Figure 12A, *p* < 0.01), including the cytosol (Figure 12B, *p* < 0.001) and the nucleus (Figure 12C, *p* < 0.05). In addition, this figure shows that, in the presence of an ET_A_ receptor antagonist, blockade of ET_B_ only induced a significant increase at the nuclear level (Figure 12A–C). As expected, in the presence of the two receptor antagonists, superfusion with ET-1 only induced a significant increase at the nuclear level (Figure 12C, *p* < 0.001). This figure also shows that the antioxidant glutathione completely reversed the ROS level in the cytosol, and partially but significantly reduced the ROS level to that measured in the presence of the two antagonists (Figure 12C).

Our results also showed that preblockade of ET_A_ and ET_B_ receptors prevents ET-1 from inducing an increase in cytosolic but not in nuclear ROS levels.

Furthermore, these results showed that the increase in cytosolic and nuclear ROS could be reversed by increasing glutathione, which suggests that the observed increase in ROS, and more particularly at the cytosolic level, is completely due to a decrease in glutathione. This seems to be partially significantly true for the increase at the nuclear level. The reason for the partial reversal by glutathione could be due, at least in part, to the high density level of the ET_B_ receptors and the very low density of ET_A_ receptors at the nuclear level of hVSMCs [3].

### 2.4. Effect of Free Extracellular Calcium on the ET-1 and ET-1 Antagonist-Induced Increase in Cytosolic and Nuclear Resting ROS Levels in hVSMCs

**Figure 13:**
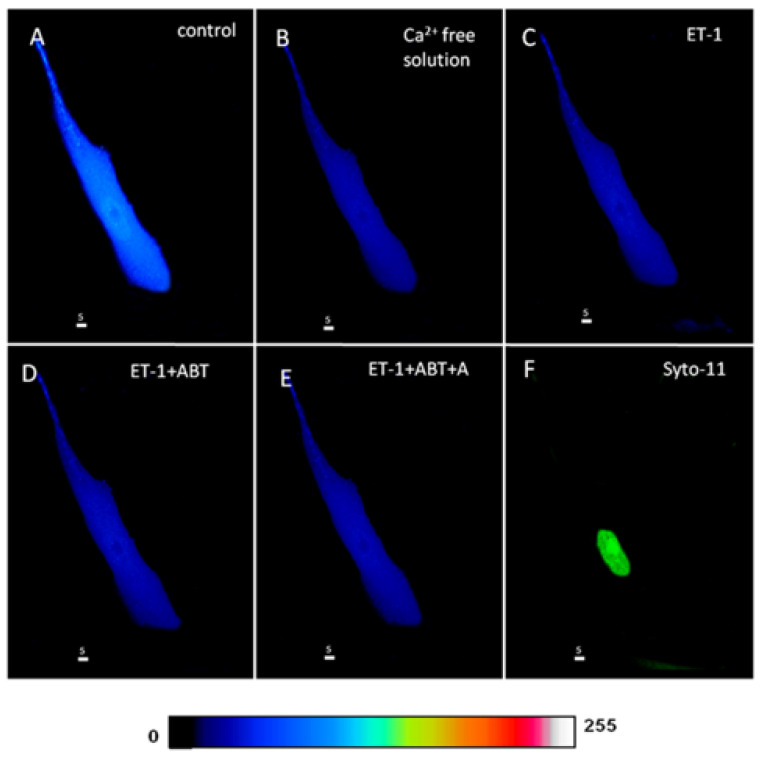
Effect of extracellular ET-1, ET_A_ antagonist ABT-627, and ET_B_ antagonist A-192621 on ROS level in a Ca^2+^-free medium in the hVSMCs. Real 3D image of hVSMCs loaded with ROS probe (Scale bars 5 µm). (**A**) Basal ROS level in the VSMC. (**B**) Removal of Ca^2+^ from the extracellular medium. Addition of ET-1 (10^−7^ M) (**C**) did not induce any changes in the ROS level. (**D**) Blocking ET_A_ receptors using its antagonist ABT-627 also did not affect the ROS level in the cell. (**E**) The addition of A-192621, an ET_B_ antagonist, did not lead to any changes in the ROS concentration in the cell. (**F**) Staining of the nucleus by the live nucleic acid stain Syto-11. The pseudocolor scale illustrates fluorescence intensity corresponding to an arbitrary scale from 0 nm to 255 nm, where 0 represents the absence of fluorescence (black), and 255 represents the maximum fluorescence (white).

**Figure 14:**
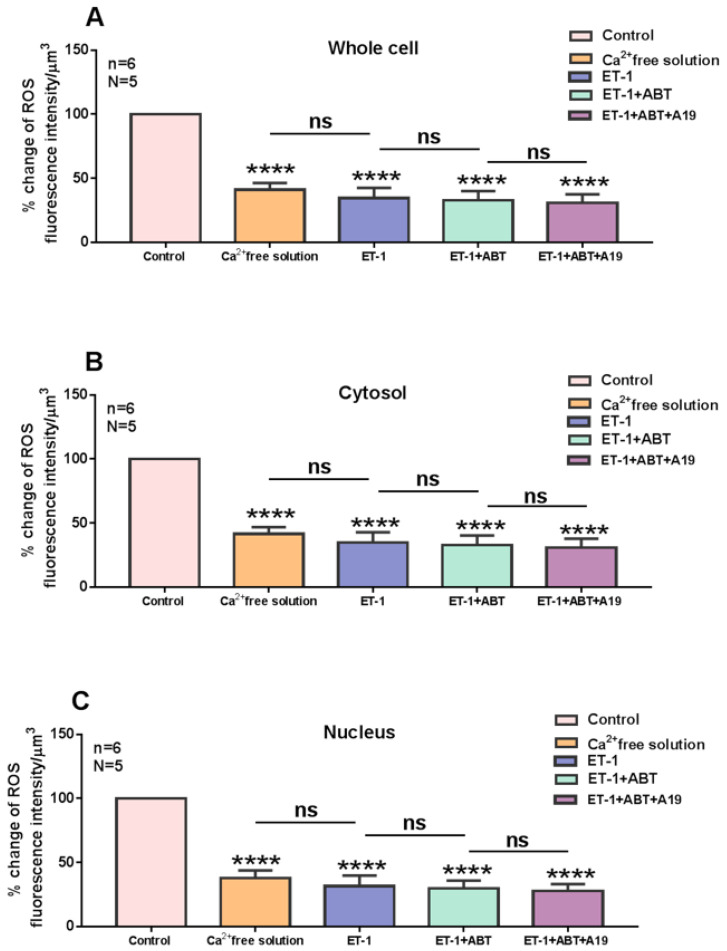
Effect of extracellular ET-1, ET_A_ receptor antagonist ABT-627, and ET_B_ antagonist A-192621 on ROS level in VSMCs in a Ca^2+^-free medium. Effect of ET-1, ET_A_ receptor antagonist ABT-627, and ET_B_ antagonist A-192621 on ROS level in a medium free of Ca^2+^ in (**A**) whole cell, (**B**) cytosol, and (**C**) nucleus of VSMCs. Values are expressed as mean ± SEM. N denotes the number of different experiments, and n denotes the number of cells. **** *p* < 0.0001, ns: not significant.

This experiment was designed to verify if the ET-1 and ET-1 antagonists induced an increase in cytosolic and nuclear Ca^2+^, mediated via an increase in Ca^2+^ influx in hVSMCs. In other terms, if the effect on ROS is Ca^2+^ dependent. Figure 13 shows an example, and Figure 14 summarizes the results. As shown in Figure 13, superfusion with Ca^2+^-free solution decreases the apparent ROS level (Figure 13B), and this decrease was highly significant (*p* < 0.001) throughout the cell, including the cytosol and the nucleus (Figure 14A–C). As Figure 13 and Figure 14 show, in the absence of Ca^2+^, neither ET-1 nor its receptor antagonists could induce any effect on ROS level.

This result clearly indicates that at least a part of the resting ROS level depends on Ca^2+^ entry through the resting R-type Ca^2+^ channel. In addition, this result indicates that the effect observed in the presence of ET-1 and ET-1 antagonists is mediated via stimulation of Ca^2+^ influx through the R-type Ca^2+^ channel. Since only Nox5 activity depends on Ca^2+^ levels, it is possible that the increase in ROS from either the blockade of ET-1 receptors or the activation of a receptor insensitive to ET_A_ and ET_B_ receptor antagonists could be due to Nox5 activation. Since the decrease in extracellular Ca^2+^ induces a decrease in intracellular ROS resting level in hVSMCs, it is possible to postulate that Nox5 basal activity highly contributes to determining the resting level of ROS. Since ROS was found to be highly present at the nuclear level in hVSMCs, this type of Nox may contribute significantly to nuclear ROS homeostasis.

## 3. Discussion

Basal ROS level plays an important role in the physiology and pathology of the cardiovascular system. This basal level depends on cell type and sex. Although the effect of vasoactive circulating factors on ROS and Ca^2+^ is well covered in the literature, we know very little concerning the resting basal level of ROS and its relation to the basal resting level of intracellular Ca^2+^.

Our results showed that basal ROS levels are heterogeneously distributed in human VSMCs and that nuclear ROS levels are higher than cytosolic levels. This result confirms those reported earlier by our group [10]. Our results showed that in resting cells, superfusion with ET_A_ and ET_B_ receptor antagonists increased basal cytosolic and nuclear ROS levels. This result demonstrates that ET_A_ and ET_B_ receptors do possess basal activity. This basal activity could be due in part to the release of ET-1 by hVSMCs, which induces activation of these two receptor types. It is also possible that both receptors in hVSMCs are active in the absence of their ligands. In addition, our results showed that blocking the basal activity of ET_A_ and ET_B_ receptors increases cytosolic and nuclear ROS levels. This suggests that the blockade of the receptors’ basal activity occurs at both the plasma membrane and the nuclear envelope. It is also possible that the increase in nuclear ROS could be due, at least in part, to the increase in cytosolic ROS. However, this latter suggestion may contribute less to the observed increase in nuclear ROS, since our results showed that nuclear ROS levels may increase independently of increases in cytosolic ROS.

Our results also showed that, in the presence of the ET_A_ receptor antagonist, the blockade of the basal activity of the ET_B_ receptors further increased nuclear ROS levels without affecting cytosolic oxidant levels. This result suggests that the basal nuclear activity of ET_B_ receptors plays an important role in determining nuclear ROS levels. This could be supported by the fact that the ET_B_ receptor is the main type of receptor in the nuclear membrane in hVSMCs [3]. In addition, this result suggests that the increase in basal cytosolic ROS due to blockade of ET_A_ receptor basal activity is mainly due to the basal activity of sarcolemmal ET_A_ receptors, which were reported to be mainly present in the plasma membrane of hVSMCs [3]. It is still surprising that receptor blockade would induce an increase in ROS. However, this phenomenon was also observed in cardiomyocytes during resting Ca^2+^ homeostasis [48]. The increase in resting ROS (this study) and resting intracellular Ca^2+^ [48], by blocking E_TA_ or ET_B_ receptor basal activities, may suggest that the increase in resting ROS level could be due in part to the increase in resting cytosolic and nuclear Ca^2+^ reported by our group, probably via the activation of Nox5, which is known to be activated by intracellular Ca^2+^ [50,51]. The increase in resting ROS as well as resting Ca^2+^ by both ET_A_ and ET_B_ receptor antagonists could be due in part to the fact that their resting activity is controlled by a negative feedback of one receptor on the other. In other words, the ET_A_ receptor acts as a physiological antagonist of the ET_B_ receptor, and vice versa. In this situation, the blockade of the basal activity of the ET_A_ receptor would release its inhibition of the basal activity of the ET_B_ receptor. Such a phenomenon could be due in part to the stimulatory effect of Gs by ET_A_ and the inhibitory effect of Gi by ET_B_ receptors.

Our results also showed that activation of ET_A_ and ET_B_ receptors by their ligand ET-1 induced a sustained increase in cytosolic and nuclear ROS in hVSMCs. This result supports those reported by our group in human endocardial endothelium cells (hEECs) and cardiomyocytes [10]. Surprisingly, the blockade of ET_A_ receptors did not reverse the effect of ET-1 on ROS. This result suggests that the effect of ET-1 on ROS in hVSMCs is not mediated by ET_A_ receptor activation. In addition, our results showed that, in the presence of ET-1, ET_A_ blockade further increased cytosolic and nuclear ROS levels. This additional increase in intracellular ROS could be due in part to the blockade of the physiological inhibitory effect of ET_A_ receptors on ET_B_ receptors. Again, surprisingly, the blockade of ET_B_ receptors did not affect either the ET-1-induced increase in cytosolic and nuclear ROS or the further increase observed with ET_A_ receptor blockade. Since the blockade of both ET_A_ and ET_B_ did not affect the ET-1-induced increase in ROS in hVSMCs, this suggests that neither ETA nor ET_B_ receptors are implicated in the ET-1 effect on cytosolic and nuclear ROS. The absence of a further increase in ROS with ET_B_ receptor blockade in the presence of ET-1 suggests that ET_B_ receptors do not necessarily negatively regulate ET_A_ receptor activity; however, the opposite is more likely.

Furthermore, our results showed that not only did the ET_A_ and ET_B_ receptor antagonists not reverse the effect of ET-1 on ROS in hVSMCs, but they also did not prevent the increase in ROS induced by ET-1. All these results suggest that the ET-1-induced increase in ROS is not mediated by known ET-1 receptors. These results suggest, as reported earlier, the possible presence of a third type of ET-1 receptor, named ET_C_ [48]. However, this type could be a heterodimeric ETA/ETB receptor present in hVSMCs. This is very likely, since our group showed that in hVSMCs both ET_A_ and ET_B_ receptors [48], and since the heterodimerized receptor has also been reported to be present in the rat mesenteric artery [35]. Since in hVSMCs, only ET_B_ receptors are present at the nuclear membrane, it is possible to suggest that nuclear ET_B_ homodimers regulate nuclear ROS levels.

Our results also showed that the antioxidant glutathione reversed the increase in ROS induced by ET_A_ and ET_B_ receptor blockade, as well as the ET-1-induced increase in cytosolic ROS. This result suggests that the contribution of ET_A_ and ET_B_ receptor basal activity and ET-1 to the cytosolic ROS level is mediated in part by a decrease in the antioxidant glutathione. However, at the nuclear level, our results showed that glutathione only reversed the increase in nuclear ROS induced by ET-1, without affecting the increase induced by ET_A_ and ET_B_ blockade of basal activity. These results suggest that antioxidant levels do not necessarily regulate basal ROS levels. However, this antioxidant plays an important role in ET-1-induced nuclear ROS generation; this should be verified in the future.

Study Limitations: This study was done in human VSMCs of a 35-year-old woman donor. In future studies, we should use the aortas of young women as well as those of young men in order to verify any sex-dependent phenomena.

## 4. Materials and Methods

### 4.1. Vascular Smooth Muscle Cells Isolation and Culture

This procedure was conducted in accordance with the institutional review committee’s standards outlined in the Declaration of Helsinki for the use of human tissue. The aorta of a donor, supplied by Transplant Québec, was carried to the laboratory in an ice-cold sterile environment composed of HMEM (Hank’s Minimal Essential Media) (Gibco-BRL, Burlington, ON, Canada), with 5% FBS (fetal bovine serum) (Gibco-BRL, Burlington, ON, Canada) and penicillin-G-Potassium (50 IU/mL) (Ayerst, Montreal, QC, Canada) [52]. The aorta was opened longitudinally and cleansed of blood with sterile M199 medium containing 2% penicillin, 2% streptomycin, and 10% FBS. To reach the VSMC layer, the endothelial cell layer was isolated by gently scraping the luminal side of the opened aorta with a sterile scalpel blade. The denuded aorta was placed in medium M199 (Gibco-BRL, Burlington, ON, Canada) containing antibiotics and 0.1% collagenase for 15 min. Then, the VSMCs were gently scraped with a sterile scalpel blade. The cell-containing medium was collected and centrifuged for 10 min at 1000 rpm at 4 °C. Afterwards, the collagenase solution was discarded, and the remaining cell pellet was resuspended in M199 culture medium containing 2% penicillin, 2% streptomycin, and 10% FBS [52].

For studies on freshly isolated cells, single cells were left to adhere to a 25 mm-diameter glass slide that fit in a (35 mm × 10 mm) cell culture dish for 1 h [52]. Subsequently, the cells were incubated at 37 °C, 95% air, and 5% CO_2_ for at least 24 h. To provide primary cell lines, cells were grown in culture flasks (Gibco-BRL, Burlington, ON, Canada) and incubated as indicated above. At confluence, cells were removed by trypsinisation (0.1% trypsin) and cultured in smooth muscle growth medium (SmGM) (Clonetics, San Diego, CA, USA) supplemented with 5% FBS [52]. Cell lines were kept frozen at –80 °C or in liquid nitrogen. They were defrosted and plated when necessary [52].

To evaluate the quality and purity of the VSMCs, tests were performed regularly using immunofluorescence and specific antibodies to detect smooth muscle α-actin. These VSMCs were shown to exhibit electrical and pharmacological properties similar to those of freshly isolated human VSMCs.

### 4.2. Confocal Microscopy and Image Analysis

#### 4.2.1. Principle of Confocal Microscopy

The optical imaging technique used in this study is confocal microscopy (BioRAD, Mississauga, ON, Canada). This technique is typically used in our laboratory because it enables us to see deep into cells and explore their subcellular structures, observe the dynamics of cellular and subcellular processes, reconstruct different types of 3D representations, record kinetics, and evaluate the presence, distribution, and dynamics of target entities. In addition to all of the above, confocal microscopy is more advantageous than conventional fluorescence microscopy [52]. In fact, it permits a non-invasive scanning of a biological specimen and the generation and 3D reconstruction of serial optical images under any angle or cutting plane. At the same time, it eliminates out-of-focus light by using an interplay between mirrors and filters within the system, which results in the generation of high-quality images with a resolution about 30% higher than that of a conventional microscope [52].

Dye-loaded cells were examined with an MRC1024 Krypton/Argon and UV laser (BioRAD, Mississauga, ON, Canada) confocal system equipped with an inverse phase epifluorescence microscope (Nikon Eclipse TE300, BioRAD, Mississauga, ON, Canada) and a 60× Nikon Oil Plan achromat objective.

To obtain the sample, the microscope lens directs an excitation laser beam, whose wavelength corresponds to the fluorescent probe in use, towards a certain point of the sample named the focal point, which lies in the focal plane [52]. The light emitted from the focal point follows the same path as that of excitation and is led through the microscope’s different mirrors and optical filters, which then converge this beam to another point named the confocal point [52]. A system of shutters, situated at the same level as the confocal point, will obstruct all incoming light stemming from points that are located above or beneath the focal plane, that is to say, out-of-focus light [52]. As a result, only light coming from the focal point will pass through the confocal point to be detected by the photomultiplier tube (the detector). This explains the unique and exceptional resolution of the images generated by the confocal microscope [52].

#### 4.2.2. Serial Scan in 3 Dimensions

The confocal microscope permits the collection of optical slices of a biological sample, or in our case, a cell, by changing the focal plane in which the cell is being visualized. When one line in the plane is scanned, a mirror directs the laser beam one step forward in the Y-axis, and another line is scanned, until the whole focal plane is scanned line by line. Then the system moves the laser beam one step in the *Z*-axis to a new focal plane, and the same process takes place. Thus, when the focal plane is changed, a sequence of X–Y images at different Z positions is produced [52]. This series of images is then used to generate a 3-D reconstruction of the cell being visualized. The benefit of the serial scanning is that only one plane is illuminated at a time, and the fluorescence collected from that plane is filtered through the confocal point of the system. This allows the removal of all fluorescence coming from out-of-focus areas [52]. Again, the serial scan is fundamental because in order to produce a real 3-D reconstruction of the cell, it has to be scanned from side to side, in lines constituting planes from top to bottom [52]. Afterward, the section series taken for the cell is captured by a photodetector, digitalized and saved. This series of sections can then be presented either as 2-D or 3-D reconstructions using various angles of rotation and inclination, as well as a variety of cutting planes by means of the image processing software “Imagespace”v3.20 [52]. This 3D reconstruction of the cell is then used to measure basal-level fluorescence intensity and the cellular response after the addition of different agents [52].

#### 4.2.3. Settings of the Confocal Microscope

One of the main elements in confocal microscopy is the setting of optimal parameter conditions. These parameters are determined prior to initiating any experiment and are captured and conserved for each specific fluorescent probe. The 568 and 647 nm Argon Ion or Krypton/Argon laser is directed to the sample via a 510 nm primary dichroic filter and is attenuated with a 1–3% neutral-density filter to reduce the photobleach. Subsequently, the beam is filtered through the corresponding barrier filters for each wavelength. The pinhole size is set at 50 μm. The image size is set at 512 × 512 pixels with a pixel size of 0.34 μm. The step size is set to 0.5 µm [52]. To optimize and validate fluorescence intensity measurements, laser line intensity, photometric gain, photomultiplier tube (PMT) settings, and filter attenuation are kept unchanged throughout the experimental procedures.

#### 4.2.4. Volume Rendering and 3D Reconstruction

Scanned images were transferred to a Silicon Graphics O2 analysis station (Sunnyvale, CA, USA) equipped with Molecular Dynamics ImageSpace analysis and Volume Workbench software modules [52]. 3D image reconstruction was performed from unfiltered serial sections. All 3D reconstructions are presented as ‘maximum intensity’ projections [52]. The ‘Maximum intensity’ format presents the highest intensities from all sections of a serial scan in a single image, thereby generating high-contrast images. This type of projection is sensitive to noise but is more convenient than other 3D reconstruction formats because it depicts more details. The images are presented as pseudocolored representations corresponding to an intensity scale from 0 to 255, where 0 intensity (black) indicates the absence of fluorescence and 255 (white) indicates maximal fluorescence intensity. For this reason, pseudo-scales are included with all images illustrating pseudo-colored representations of intensity levels.

### 4.3. Detection of Ions and Cell Structures with Fluorescent Probes

#### 4.3.1. Loading of Cells with ROS Probe

VSMCs were cultured on 25 mm diameter glass coverslips that fit a 1 mL bath chamber. Prior to loading, the cells were washed 3 times with 2 mL of a balanced Tyrode’s salt solution containing 5 mM HEPES, 136 mM NaCl, 2.7 mM KCl, 1 mM MgCl2, 1.9 mM CaCl2, 5.6 mM glucose, and 0.1% bovine serum albumin (BSA). The pH of this solution (with or without BSA) was adjusted to 7.4 with Tris base, and the osmolarity was regulated to 310 mOsm with sucrose using a Digimatic Osmometer (Advanced Instruments Inc., Norwood, MA, USA). The carboxy H2DCEF-DA “carboxy Dichlorodihydrofluorescin Diacetate” (Invitrogen, Burlington, ON, Canada) is a probe that fluoresces once it is oxidized. The probe was diluted in Tyrode-BSA from a 0.1 M stock in dimethyl sulfoxide (DMSO) to a final concentration of 200 µM. Cells were loaded by placing the coverslips cell-side down on a sheet of Parafilm stretched over a glass plate containing a 100 μL drop of the diluted probe. The cells were incubated for 30–60 min in the dark at 37 °C in a humidified environment to prevent evaporation. The inverted coverslip method was used for two reasons: first, because it helps conserve a large amount of probe, especially at high concentrations, and, above all, because using smaller aliquots of concentrated stock solution needed to prepare the final probe concentration reduces the percentage of DMSO in the incubation medium. In fact, it has been frequently remarked in our laboratory that cells would undergo blebbing and photobleaching when the final DMSO concentration was greater than 0.6% [52]. After the loading period, coverslips were recovered, and the cells were washed twice with Tyrode-BSA buffer and twice in Tyrode buffer alone.

#### 4.3.2. Loading of Cells with Glutathione Probe

The glutathione fluorescent probe Cell Tracker Green CMFDA (Life Technologies, Burlington, ON, Canada) was diluted in Tyrode-BSA from a 10 mM stock in dimethyl sulfoxide (DMSO) to a final concentration of 10 µM. The cells were loaded with the probe for 30–60 min in the same way as described above for the ROS probe.

#### 4.3.3. Nuclear Straining

At the end of each experiment, the nucleus was marked with 100 nM of live cell nucleic acid stain Syto 11 (Molecular Probes, Eugene, OR, USA). Serial Z-axis optical scans were taken directly after development of the stain (which takes about 8–10 min) while maintaining positioning, number of sections, and step size identical to those used throughout the experiment. Nuclear labeling is essential because it permits the extraction of the nuclear area from the cytoplasm by setting a lower intensity threshold filter to confine relevant pixels. Next, a 3-D binary image series of the nuclear volume was generated for each cell using the exact same x, y, and z set planes as those used during the experiment. By then applying these binary image patterns of the nucleus to the same cell but labeled with the corresponding probe, a new 3-D projection was created depicting fluorescence intensity levels exclusively within the nucleus. Hence, by ‘removing’ the nucleus from the surrounding cytoplasm, measurements of the mean fluorescence intensity values in the entire nuclear volume, while eliminating possible contribution of the perinuclear region, were taken. This is how fluorescence intensities were measured in the entire volume of both compartments (cytosol and nucleus), distinctly to provide 3D information [52].

### 4.4. Administration of the Different Substances

All experiments started out the same way. Cells were put under the microscope, and were scanned after 3–5 min in order to determine the basal level of ROS or glutathione (depending on the experiment) in those cells. To test the cell response to the pharmacological stimuli, cells were scanned at different intervals after the addition of ET-1 (10^−7^ M) (American Peptide Company, Sunnyvale, CA, USA) or ABT-627 (10^−7^ M) and A-192621 (10^−7^ M) (donation from Dr. Pedro D’Orleans Juste), or glutathione (10 mM), until a steady state situation is reached. As mentioned earlier, at the end of the experiment, the nucleus was stained with the Syto-11 probe.

### 4.5. Statistics

Fluorescence intensity values are expressed as means ± SEM of N different experiments on n number of cells. Statistical significance was determined using the ANOVA repeated measures test for matched values, followed by a Bonferroni multiple comparison test to assess statistical significance of the results. *p*-values of less than 0.05 were considered significant.

## 5. Conclusions

Finally, our results showed that the resting ROS level is determined, at least in part, by intracellular Ca^2+^ levels at both the cytosolic and nuclear levels. In addition, the resting level of ROS appears to be modulated by the receptor’s resting activity, such as ET-1. One important aspect of our findings is that the basal activity of heterodimerized receptors, such as ET_A_-ET_B,_ could mediate the resting level of ROS. We previously showed that ET_A_-ET_B_ receptors are heterodimerized in human VSMCs and that resting ET-1 binds to this complex [3]. This may explain, in part, the absence of effect of ET_A_ and ET_B_ receptor antagonists in the treatment of hypertension and the beneficial effect of the dual ET_A_-ET_B_ antagonist Aprocitentan in the treatment of resistant hypertension [53,54,55,56]. The modulation of ROS level by ET-1 and its receptors is mediated via Ca^2+^ entry through probably the resting steady-state R-type Ca^2+^ channel.

## Data Availability

The original contributions presented in this study are included in the article. Further inquiries can be directed to the corresponding author.

## References

[1] Yanagisawa M., Kurihara H., Kimura S., Goto K., Masaki T. (1988). A novel peptide vasoconstrictor, endothelin, is produced by vascular endothelium and modulates smooth muscle Ca^2+^ channels. J. Hypertens..

[2] Clarke J.G., Benjamin N., Larkin S.W., Webb D.J., Davies G.J., Maseri A. (1989). Endothelin is a potent long-lasting vasoconstrictor in men. Am. J. Physiol..

[3] Bkaily G., Avedanian L., Al-Khoury J., Provost C., Nader M., D’Orléans-Juste P., Jacques D. (2011). Nuclear membrane receptors for ET-1 in cardiovascular function. Am. J. Physiol. Regul. Integr. Comp. Physiol..

[4] Yanagisawa M., Kurihara H., Kimura S., Tomobe Y., Kobayashi M., Mitsui Y., Yazaki Y., Goto K., Masaki T. (1988). A novel potent vasoconstrictor peptide produced by vascular endothelial cells. Nature.

[5] Kanai S.M., Clouthier D.E. (2023). Endothelin signaling in development. Development.

[6] Brunner F., Bras-Silva C., Cerdeira A.S., Leite-Moreira A.F. (2006). Cardiovascular endothelins: Essential regulators of cardiovascular homeostasis. Pharmacol. Ther..

[7] Kedzierski R.M., Yanagisawa M. (2001). Endothelin system: The double-edged sword in health and disease. Annu. Rev. Pharmacol. Toxicol..

[8] Hosoda K., Nakao K., Hiroshi A., Suga S.-i., Ogawa Y., Mukoyama M., Shirakami G., Saito Y., Nakanishi S., Imura H. (1991). Cloning and expression of human endothelin-1 receptor cDNA. FEBS Lett..

[9] Molenaar P., O’Reilly G., Sharkey A., Kuc R.E., Harding D.P., Plumpton C., Gresham G.A., Davenport A.P. (1993). Characterization and localization of endothelin receptor subtypes in the human atrioventricular conducting system and myocardium. Circ. Res..

[10] Provost C., Choufani F., Avedanian L., Bkaily G., Gobeil F., Jacques D. (2010). Nitric oxide and reactive oxygen species in the nucleus revisited. Can. J. Physiol. Pharmacol..

[11] Merlen C., Farhat N., Luo X., Chatenet D., Tadevosyan A., Villeneuve L.R., Gillis M.A., Nattel S., Thorin E., Fournier A. (2013). Intracrine endothelin signaling evokes IP3-dependent increases in nucleoplasmic Ca^2+^ in adult cardiac myocytes. J. Mol. Cell. Cardiol..

[12] Arai H., Hori S., Aramori I., Ohkubo H., Nakanishi S. (1990). Cloning and expression of a cDNA encoding an endothelin receptor. Nature.

[13] Hori S., Komatsu Y., Shigemoto R., Mizuno N., Nakanishi S. (1992). Distinct tissue distribution and cellular localization of two messenger ribonucleic acids encoding different subtypes of rat endothelin receptors. Endocrinology.

[14] Titus A., Marappa-Ganeshan R. (2025). Physiology, Endothelin. StatPearls.

[15] Takayanagi R., Kitazumi K., Takasaki C., Ohnaka K., Aimoto S., Tasaka K., Ohashi M., Nawata H. (1991). Presence of non-selective type of endothelin receptor on vascular endothelium and its linkage to vasodilation. FEBS Lett..

[16] Mikhail M., Vachon P.H., D’Orleans-Juste P., Jacques D., Bkaily G. (2017). Role of endothelin-1 and its receptors, ET_A_ and ET_B_, in the survival of human vascular endothelial cells. Can. J. Physiol. Pharmacol..

[17] Moreland S., McMullen D.M., Delaney C.L., Lee V.G., Hunt J.T. (1992). Venous smooth muscle contains vasoconstrictor ETB-like receptors. Biochem. Biophys. Res. Commun..

[18] White D.G., Cannon T.R., Garratt H., Mundin J.W., Sumner M.J., Watts I.S. (1993). Endothelin ET_A_ and ET_B_ receptors mediate vascular smooth-muscle contraction. J. Cardiovasc. Pharmacol..

[19] Williams D.L., Jones K.L., Pettibone D.J., Lis E.V., Clineschmidt B.V. (1991). Sarafotoxin S6c: An agonist which distinguishes between endothelin receptor subtypes. Biochem. Biophys. Res. Commun..

[20] Montezano A.C., Burger D., Paravicini T.M., Chignalia A.Z., Yusuf H., Almasri M., He Y., Callera G.E., He G., Krause K.H. (2010). Nicotinamide adenine dinucleotide phosphate reduced oxidase 5 (Nox5) regulation by angiotensin II and endothelin-1 is mediated via calcium/calmodulin-dependent, Rac-1-independent pathways in human endothelial cells. Circ. Res..

[21] Inostroza-Nieves Y., Bou S., Alvarado J., Capo-Ruiz D., Garcia J., Moliere J.P., Arenas C.P. (2025). Endothelin-1 triggers oxidative stress and cytokine release in human microglia cells through ETRB-dependent mechanisms. Front. Cell. Neurosci..

[22] Montezano A.C., Touyz R.M. (2012). Reactive oxygen species and endothelial function—Role of nitric oxide synthase uncoupling and Nox family nicotinamide adenine dinucleotide phosphate oxidases. Basic Clin. Pharmacol. Toxicol..

[23] Wedgwood S., McMullan D.M., Bekker J.M., Fineman J.R., Black S.M. (2001). Role for endothelin-1-induced superoxide and peroxynitrite production in rebound pulmonary hypertension associated with inhaled nitric oxide therapy. Circ. Res..

[24] Habib N.G., Adhia A., Lopez D., Sandros M., Guichard P. (2026). Selection of Endothelin Receptor Antagonists in the Treatment of Pulmonary Arterial Hypertension: A Comprehensive Narrative Review. Adv. Ther..

[25] Loomis E.D., Sullivan J.C., Osmond D.A., Pollock D.M., Pollock J.S. (2005). Endothelin Mediates Superoxide Production and Vasoconstriction through Activation of NADPH Oxidase and Uncoupled Nitric-Oxide Synthase in the Rat Aorta. J. Pharmacol. Exp. Ther..

[26] Meyer M.R., Barton M., Prossnitz E.R. (2014). Functional heterogeneity of NADPH oxidase-mediated contractions to endothelin with vascular aging. Life Sci..

[27] Ruef J., Moser M., Kubler W., Bode C. (2001). Induction of endothelin-1 expression by oxidative stress in vascular smooth muscle cells. Cardiovasc. Pathol..

[28] Dong F., Zhang X., Wold L.E., Ren Q., Zhang Z., Ren J. (2005). Endothelin-1 enhances oxidative stress, cell proliferation and reduces apoptosis in human umbilical vein endothelial cells: Role of ETB receptor, NADPH oxidase and caveolin-1. Br. J. Pharmacol..

[29] Wedgwood S., Black S.M. (2005). Endothelin-1 decreases endothelial NOS expression and activity through ET_A_ receptor-mediated generation of hydrogen peroxide. Am. J. Physiol. Lung Cell. Mol. Physiol..

[30] Serafim K.G.G., Navarro S.A., Zarpelon A.C., Pinho-Ribeiro F.A., Fattori V., Cunha T.M., Alves-Filho J.C., Cunha F.Q., Casagrande R., Verri W.A. (2015). Bosentan, a mixed endothelin receptor antagonist, inhibits superoxide anion-induced pain and inflammation in mice. Naunyn-Schmiedeberg’s Arch. Pharmacol..

[31] Gupta S.K., Saxena A., Singh U., Arya D.S. (2005). Bosentan, the mixed ET_A_-ET_B_ endothelin receptor antagonist, attenuated oxidative stress after experimental myocardial ischemia and reperfusion. Mol. Cell. Biochem..

[32] Singh A.D., Amit S., Kumar O.S., Rajan M., Mukesh N. (2006). Cardioprotective Effects of Bosentan, a Mixed Endothelin Type A and B Receptor Antagonist, during Myocardial Ischaemia and Reperfusion in Rats. Basic Clin. Pharmacol. Toxicol..

[33] Rafikova O., Rafikov R., Kumar S., Sharma S., Aggarwal S., Schneider F., Jonigk D., Black S.M., Tofovic S.P. (2013). Bosentan inhibits oxidative and nitrosative stress and rescues occlusive pulmonary hypertension. Free Radic. Biol. Med..

[34] Mazzuca M.Q., Khalil R.A. (2012). Vascular endothelin receptor type B: Structure, function and dysregulation in vascular disease. Biochem. Pharmacol..

[35] Kapsokalyvas D., Schiffers P.M., Maij N., Suylen D.P., Hackeng T.M., van Zandvoort M.A., De Mey J.G. (2014). Imaging evidence for endothelin ETA/ETB receptor heterodimers in isolated rat mesenteric resistance arteries. Life Sci..

[36] Yatawara A., Wilson J.L., Taylor L., Polgar P., Mierke D.F. (2013). C-terminus of ETA/ETB receptors regulate endothelin-1 signal transmission. J. Pept. Sci..

[37] Luo M., Anderson M.E. (2013). Mechanisms of altered Ca^2+^ handling in heart failure. Circ. Res..

[38] Bkaily G., Avedanian L., Al-Khoury J., Chamoun M., Semaan R., Jubinville-Leblanc C., D’Orléans-Juste P., Jacques D. (2015). Nuclear membrane R-type calcium channels mediate cytosolic ET-1-induced increase of nuclear calcium in human vascular smooth muscle cells. Can. J. Physiol. Pharmacol..

[39] Bkaily G., Jacques D. (2023). Calcium Homeostasis, Transporters, and Blockers in Health and Diseases of the Cardiovascular System. Int. J. Mol. Sci..

[40] Ajanel A., Andrianova I., Kowalczyk M., Menendez-Perez J., Bhatt S.R., Portier I., Boone T.C., Ballard-Kordeliski A., Kosaka Y., Chaudhuri D. (2025). Mitochondrial Calcium Uniporter Regulates ITAM-Dependent Platelet Activation. Circ. Res..

[41] Bkaily G., Najibeddine W., Jacques D. (2019). Increase of NADPH oxidase 3 in heart failure of hereditary cardiomyopathy. Can. J. Physiol. Pharmacol..

[42] Yan Y., Wei C.L., Zhang W.R., Cheng H.P., Liu J. (2006). Cross-talk between calcium and reactive oxygen species signaling. Acta Pharmacol. Sin..

[43] Sousa S.C., Maciel E.N., Vercesi A.E., Castilho R.F. (2003). Ca^2+^-induced oxidative stress in brain mitochondria treated with the respiratory chain inhibitor rotenone. FEBS Lett..

[44] Harada N., Himeno A., Shigematsu K., Sumikawa K., Niwa M. (2002). Endothelin-1 binding to endothelin receptors in the rat anterior pituitary gland: Possible formation of an ET_A_-ET_B_ receptor heterodimer. Cell. Mol. Neurobiol..

[45] Himeno A., Shigematsu K., Taguchi T., Niwa M. (1998). Endothelin-1 binding to endothelin receptors in the rat anterior pituitary gland: Interaction in the recognition of endothelin-1 between ET_A_ and ET_B_ receptors. Cell. Mol. Neurobiol..

[46] Dai X., Galligan J.J. (2006). Differential trafficking and desensitization of human ET_A_ and ET_B_ receptors expressed in HEK 293 cells. Exp. Biol. Med..

[47] Gregan B., Jurgensen J., Papsdorf G., Furkert J., Schaefer M., Beyermann M., Rosenthal W., Oksche A. (2004). Ligand-dependent differences in the internalization of endothelin A and endothelin B receptor heterodimers. J. Biol. Chem..

[48] Bkaily G., Massaad D., Choufani S., Jacques D., D’Orleans-Juste P. (2002). Role of endothelin-1 receptors in the sarcolemma membrane and the nuclear membrane in the modulation of basal cytosolic and nuclear calcium levels in heart cells. Clin. Sci..

[49] Ahmarani L., Avedanian L., Al-Khoury J., Perreault C., Jacques D., Bkaily G. (2013). Whole-cell and nuclear NADPH oxidases levels and distribution in human endocardial endothelial, vascular smooth muscle, and vascular endothelial cells. Can. J. Physiol. Pharmacol..

[50] Griendling K.K., Camargo L.L., Rios F.J., Alves-Lopes R., Montezano A.C., Touyz R.M. (2021). Oxidative Stress and Hypertension. Circ. Res..

[51] Touyz R.M., Briones A.M., Sedeek M., Burger D., Montezano A.C. (2011). NOX isoforms and reactive oxygen species in vascular health. Mol. Interv..

[52] Bkaily G., Pothier P., D’Orléans-Juste P., Simaan M., Jacques D., Jaalouk D., Belzile F., Hassan G., Boutin C., Haddad G. (1997). The use of confocal microscopy in the investigation of cell structure and function in the heart, vascular endothelium and smooth muscle cells. Mol. Cell. Biochem..

[53] Sidhu J.K., Chopra D., Goyal A., Lhamo Y. (2026). A New Drug for Resistant Hypertension: Aprocitentan. Curr. Hypertens. Rev..

[54] Fields E., Schiffrin E.L. (2025). Aprocitentan: A new horizon in the treatment of hypertension. Expert Opin. Pharmacother..

[55] Nguyen T., Lin H., Tint D., Dima L., Wang Z.A. (2025). Aprocitentan: The First Endothelin Receptor Antagonist for Resistant Hypertension. Am. J. Ther..

[56] Kumbhare M., Ide B.R., Shaikh A., Gode H., Pagere N. (2025). Aprocitentan in Resistant Hypertension: Mechanistic Insights, Clinical Evidence, and Future Directions. Chin. J. Appl. Physiol..

